# Molecular Typing and Clinical Characteristics of Synchronous Multiple Primary Colorectal Cancer

**DOI:** 10.1001/jamanetworkopen.2022.43457

**Published:** 2022-11-23

**Authors:** Yandong Zhao, Jingjing Wu, Fengyun Pei, Yanxiang Zhang, Shaomei Bai, Lishuo Shi, Xiang Zhang, Jingjiao Ma, Ximeng Zhao, Tonghui Ma, Jianping Wang, Meijin Huang, Xinjuan Fan, Jun Huang

**Affiliations:** 1 Department of Pathology, The Sixth Affiliated Hospital, Sun Yat-sen University, Guangzhou, China; 2 Department of Colorectal Surgery, The Sixth Affiliated Hospital, Sun Yat-sen University, Guangzhou, China; 3 Jichenjunchuang Clinical Laboratory, Hangzhou, China; 4 Clinical Research Center, The Sixth Affiliated Hospital, Sun Yat-sen University, Guangzhou, China; 5 Guangdong Provincial Key Laboratory of Colorectal and Pelvic Floor Diseases, The Sixth Affiliated Hospital, Sun Yat-sen University, Guangzhou, China; 6 Guangdong Institute of Gastroenterology, Guangzhou, China

## Abstract

**Importance:**

Synchronous multiple primary colorectal cancer (sMPCC) is clinically rare, but its incidence has increased over the past decade. However, little is known about the molecular and clinical features of sMPCC, which may differ from those of single primary colorectal cancer (SPCRC).

**Objective:**

To evaluate the clinical characteristics and pathogenic variations in lesions and the molecular typing of sMPCC.

**Design, Setting, and Participants:**

From November 2012 to April 2021, patients with colorectal cancer (CRC) treated at the Sixth Affiliated Hospital of Sun Yat-sen University were enrolled in this cohort study. Follow-up ended on January 31, 2022.

**Main Outcomes and Measures:**

The primary outcome was mismatch repair (MMR) status of each lesion in all patients examined using immunohistochemistry (IHC). Microsatellite instability (MSI) and tumor mutation burden (TMB) were also calculated.

**Results:**

A total of 13 276 patients with CRC were enrolled, and 239 patients with sMPCC (mean [SD] age, 63.3 [12.2] years; 173 men [72.4%]) with available clinical data were evaluated. Seventy-eight patients with sMPCC and 94 with SPCRC also underwent next-generation sequencing (NGS)–based molecular testing. The deficient MMR (dMMR)/MSI-H frequencies in sMPCC were significantly higher than those in SPCRC, which was confirmed by both IHC (50 of 239 patients vs 872 of 13 037 patients) and NGS (17 of 78 patients vs 5 of 94 patients). According to the MMR/MSI status of different lesions in patients with sMPCC, they were further divided into 3 subgroups: all dMMR/MSI-H, dMMR/MSI-H and proficient MMR (pMMR)/microsatellite stability (MSS), and all pMMR/MSS. The *EGFR* and *PIK3CA* variants were more common, whereas *TP53* variants were less prevalent in patients with sMPCC than in those with SPCRC. Moreover, higher tumor mutation burden was associated with higher MSI in patients with sMPCC rather than in those with SPCRC.

**Conclusions and Relevance:**

In this cohort study of sMPCC, the incidence of dMMR/MSI-H in patients with sMPCC was significantly higher than that in patients with SPCRC. These findings suggest that sMPCC can be classified into 3 subgroups according to the MMR/MSI status of each lesion, which might be applied to guide personalized therapies for better disease management.

## Key Points

**Question:**

What are the molecular characteristics and clinical features of synchronous multiple primary colorectal cancer (sMPCC)?

**Findings:**

This cohort study of 239 patients with sMPCC found that the deficient mismatch repair (dMMR)/microsatellite instability–high (MSI-H) frequencies in sMPCC were significantly higher than those in single primary colorectal cancer. The MMR/MSI status of each lesion might be different in sMPCC and can be classified into 3 subgroups: all dMMR/MSI-H, dMMR/MSI-H and proficient MMR (pMMR)/microsatellite stability (MSS), and all pMMR/MSS.

**Meaning:**

These findings suggest that sMPCC can be classified into subgroups according to the MMR/MSI status of each lesion, which might be applied to guide personalized therapies for better disease management.

## Teaser

This cohort study evaluates the molecular characteristics and clinical features of synchronous multiple primary colorectal cancer.

## Introduction

Synchronous multiple primary colorectal cancer (sMPCC) is a relatively rare colorectal cancer (CRC) that refers to the simultaneous occurrence of 2 or more independent primary malignant tumors in the colon or rectum of the same patient. According to recent reports,^1,2^ despite the slightly lower prevalence of CRC in China than in the US, the annual incidence and mortality of CRC in China has increased steadily since 2000, and the incidence rate of sMPCC is also increasing. Although the incidence rate varies slightly in different regions, a meta-analysis^3,4^ showed that the overall incidence rate of sMPCC in all CRCs was approximately 1.1% to 8.1%. The relatively low incidence rate has led to a lack of large-scale research on sMPCC. Most studies investigating sMPCC have focused on the clinical characteristics and epidemiology of small samples.^4,5^ Only a few studies have provided genome-wide characterization of sMPCC,^6,7^ and, to our knowledge, there are no integrated studies on the clinical phenotype and molecular characteristics of sMPCC.

Moreover, with the progress of immunotherapy in microsatellite instability–high (MSI-H) CRC, the mismatch repair (MMR)/MSI status plays an important role in CRC treatment. It remains unclear whether the MMR/MSI status of different sMPCC lesions is consistent.

In April 2020, we treated a patient in their 50s with sMPCCs and multiple liver metastases. The patient had 3 tumors located in the ascending colon (T3N2M1), transverse colon (T3N0M1), and upper rectum (T4aN2M1). Genomic sequencing of the rectal lesion revealed that the patient had MSI-H status. Therefore, after fully evaluating the condition and consulting with the patient, we performed a conversion strategy with 6 courses of programmed death (PD)–1 blockade (200 mg/dose) combined with mFOLFOX6 for 2 weeks between courses. The patient’s symptoms were alleviated rapidly, and laparoscopic resection of the whole colon and upper rectum was performed as planned without delay. Remarkably, all gastrointestinal lesions and lymph nodes in the patient had a pathological complete response. The patient received 3 additional doses of PD-1 blockade therapy (200 mg/dose) after surgery. During follow-up, routine radiological examinations, including computed tomography, magnetic resonance imaging, and contrast-enhanced ultrasonography, proved that the patient had maintained no evidence of disease status for more than 18 months. On the basis of this case, we focused on evaluating the molecular typing of sMPCC by MMR and MSI status and pathogenic variations of sMPCC. We performed a large-scale, retrospective cohort study of sMPCC to evaluate the clinical characteristics and pathogenic variations in each lesion and performed molecular typing in sMPCC for the first time, to our knowledge.

## Methods

### Patients

All procedures performed in studies involving human participants were in accordance with the ethical standards of the institutional and/or national research committee and with the 1964 Declaration of Helsinki^8^ and its later amendments or comparable ethical standards. This cohort study followed the Strengthening the Reporting of Observational Studies in Epidemiology (STROBE) reporting guideline for cohort studies, and the protocol was approved by ethics committee of medical research of the Sixth Affiliated Hospital of Sun Yat-sen University. All the patients were fully informed and signed the informed consent form.

According to the Warren and Gates criteria, the detailed criteria of sMPCC are as follows: (1) pathologically confirmed as adenocarcinoma, (2) separated from one another, (3) exclusion of metastases from the primary CRC, and (4) diagnosis together or within 6 months of each other.^3^ From November 2012 to April 2021, after excluding patients whose clinical data were incomplete or lost to follow-up, patients with CRC, including patients with sMPCC who underwent surgery at the Sixth Affiliated Hospital of Sun Yat-sen University, were included. We conducted a retrospective single-center study of all eligible patients with sMPCC. Each lesion in patients with CRC underwent pathological examination and immunohistochemistry (IHC).

The treatment and baseline information of each patient were collected using a medical record system. During the study period, follow-up methods included telephone interviews, outpatient examinations, and readmission. Follow-up ended on January 31, 2022, or when death occurred. All patients were initially followed up once every 3 months initially after the operation and then every 6 months from the third year to the fifth year if there was no recurrence. Physical examination and serum carcinoembryonic antigen and carbohydrate antigen 19-9 tests were regularly performed each time, and computed tomography was reexamined semiannually.

### Immunohistochemistry

The entire tumor tissue was subjected to microscopic examination, and formalin-fixed, paraffin-embedded tissue samples were obtained. Sections (4-μm thick) were stained with hematoxylin and eosin and examined. Immunohistochemical analysis was performed using monoclonal antibodies against MLH1 (MAXIM), MSH2 (LBP), MSH6 (ZSGB-BIO), and PMS2 (ZSGB-BIO), according to established protocols. Slides were incubated overnight with primary antibodies at 4 °C. Horseradish peroxidase–labeled secondary antibody from a MaxVisionTM HRP-Polymer goat anti–mouse/rabbit IHC kit (PV-6000, ZSGB-BIO) was applied, and the slides were incubated for 30 minutes at room temperature, followed by a 5-minute incubation at room temperature with 3,3′-diaminobenzidine. Finally, the sections were counterstained with hematoxylin and mounted using Permount (BIOS). The results were visualized and interpreted by 2 experienced pathologists (Y. Zhao and S. Bai).

### Sequencing and Analysis

Targeted next-generation sequencing (NGS) was performed on each lesion of 78 sMPCC with sufficient specimens using Onco PanScan (Genetron Health Co, Ltd). Briefly, total DNA from tumor tissues was extracted using a QIAamp DNA Tissue Kit (Qiagen). DNA libraries were constructed using a KAPA HTP Library Preparation Kit (Illumina), according to the manufacturer’s protocol. Quality control was performed using FastQC (version 0.11.2) and Trimmomatic (version 0.33).^9^ The generated sequences were mapped to the human genome HG19 using the BWA-MEM algorithm (version 0.7.10-r789)^10^ for both tumor and normal samples. Polymerase chain reaction duplications were marked and removed using Picard command line tools version 1.103 (Broad Institute). Base quality recalibration and local realignment processing were performed using the Genome Analysis Toolkit (GATK, version v3.1-0-g72492bb).^11^ Somatic single nucleotide variations and small insertions/deletions were identified using MuTect (version 3.1-0-g72492bb)^12^ and Strelka (version 1.0.14).^13^ The effects of variants were annotated using the Variant Effect Predictor (version 83)^14^ and Oncotator (version 1.5.1.0).^15^ Somatic copy number variants are determined using ADTEx.^16^ Structural variations were determined using CREST.^17^ MSI status was evaluated using NGS by calculating the length of 309 microsatellite sites. The tumor mutation burden (TMB) value was determined by the number of somatic single nucleotide variations and insertions/deletions per megabase of the 3 427 939-bp coding region.

### Statistical Analysis

Statistical analysis was performed using SPSS statistical software version 26.0 (IBM) and R statistical software version 4.0.4 for Windows (R Project for Statistical Computing). Two-tailed *P* < .05 was considered statistically significant. According to the data distribution characteristics, continuous variables were presented as mean (SD) or median (IQR), and categorical data were presented as numbers or percentages. Continuous variables between groups were compared using a *t *test when they followed a normal distribution; otherwise, a Mann-Whitney *U* nonparametric test was applied. Categorical variables were compared using the χ^2^ test or Fisher exact test. Survival analysis of the overall survival (OS) and progression-free survival (PFS) rates was performed using the Kaplan-Meier method and assessed using a log-rank test.

## Results

### Patients

**Figure 1.**
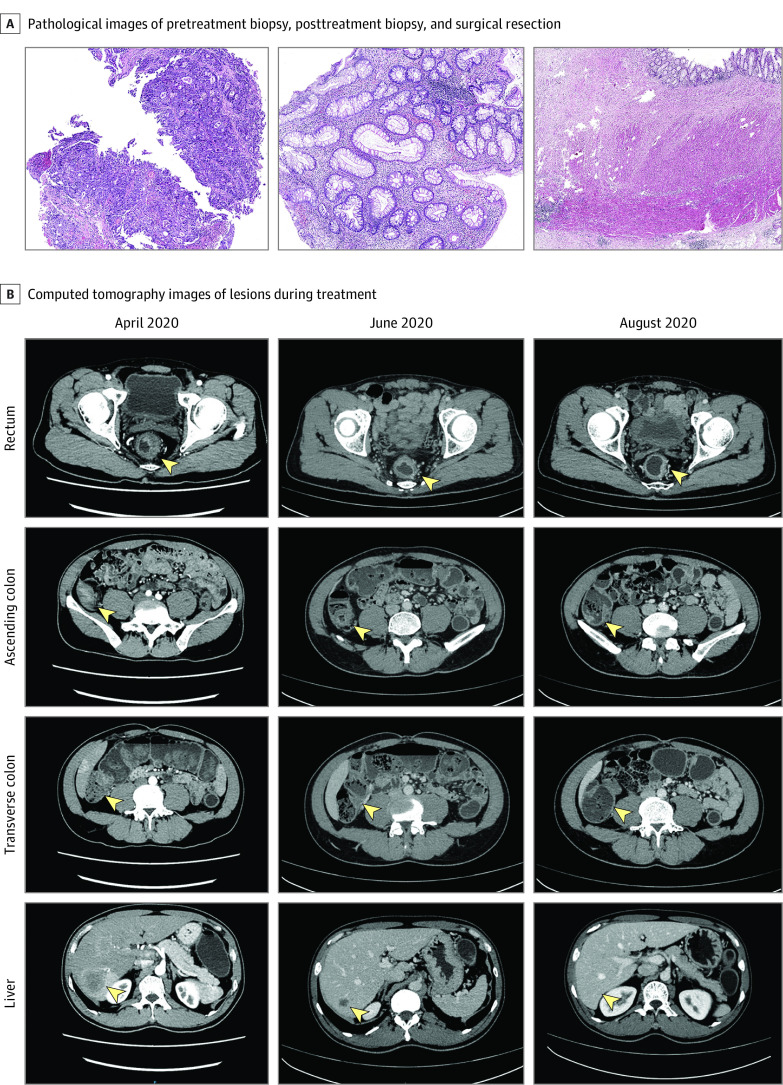
Treatment Characteristics of a Patient With Synchronous Multiple Primary Colorectal Cancer With Liver Metastases A, Pathological images of pretreatment biopsy (left, magnification, 60×), posttreatment biopsy (middle, magnification, 60×), and surgical resection (right, magnification, 20×) are shown. B, Computed tomography images of each lesion (arrowheads) during treatment are shown.

**Figure 2.**
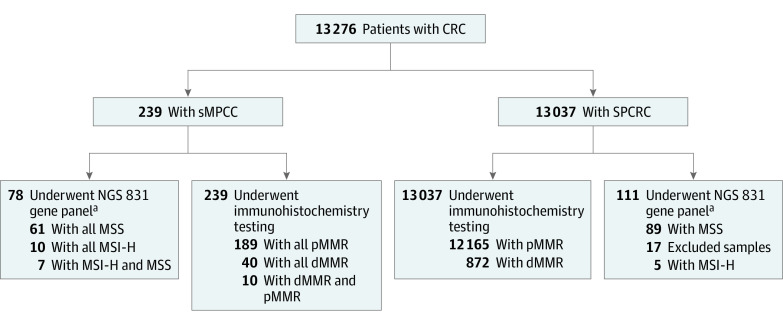
Study Flowchart CRC indicates colorectal cancer; dMMR, deficient mismatch repair; MMR, mismatch repair; MSI-H, microsatellite instability-high; MSS, microsatellite stability; NGS, next-generation sequencing; pMMR, proficient mismatch repair; sMPCC, synchronous multiple primary colorectal cancer; SPCRC, single primary colorectal cancer. ^a^Five contaminated specimens were removed.

Figure 1 and eFigure 1 in Supplement 1 show the treatment details and follow-up of the patient with sMPCC who was treated with immunotherapy. For the current cohort study, 13 276 patients with CRC were enrolled, and 239 patients with sMPCC (mean [SD] age, 63.3 [12.2] years; 173 men [72.4%]) with available clinical data were evaluated. Seventy-eight patients with sMPCC and 94 patients with single primary CRC (SPCRC) also underwent NGS-based molecular testing. Each lesion in 13 276 patients with CRC underwent pathological examination and IHC. In this cohort, 922 patients had deficient MMR (dMMR) CRC. Patient enrollment in the current study is shown in Figure 2.

### Molecular Typing of sMPCC

According to the IHC results of each lesion, the MMR status of all 239 patients with sMPCC was divided into proficient MMR (pMMR) (189 patients [79.1%]), dMMR (40 patients [16.7%]), and dMMR and pMMR (10 patients [4.2%]) groups. According to the genomic sequencing results of each lesion, the MSI status of 78 patients with sMPCC was divided into MSS (61 patients [78.2%]), MSI-H (10 patients [12.8%]), and MSI-H and MSS (7 patients [9%]) groups. MMR status was highly consistent with MSI status (75 patients [96.2%]) (eTable 1 in Supplement 1).

### Clinical Characteristics of Patients With sMPCC

An overview of the clinical trial samples included in the pooled analysis of the individual patient data is presented in Table 1. Of the 239 patients with sMPCC included in the pooled analysis, significant differences between the 3 groups were detected for age at enrollment as a continuous variable, but not as a categorical variable. No differences were observed in sex, clinical stage, number of lesions, treatment, or histologic type.

**Table 1.** Clinical Characteristics of Patients With Synchronous Multiple Primary Colorectal Cancer

Characteristic	Patients, No. (%)				*P* value
	All (n = 239)	With dMMR (n = 40)	With dMMR and pMMR (n = 10)	With pMMR (n = 189)	
Age, y					
Mean (SD)	63.3 (12.2)	53 (12.4)	66.5 (13.6)	63.3 (11.0)	<.001
Median (range)	64.0 (23.0-92.0)	54.0 (23.0-74.0)	63.5 (49.0-92.0)	65.0 (25.0-91.0)	
Sex					
Female	66 (27.6)	10 (25.0)	6 (60.0)	50 (25.5)	.06
Male	173 (72.4)	30 (75.5)	4 (40.0)	139 (73.5)	
Tumors stage					
I	26 (10.9)	4 (10.0)	1 (10.0)	21 (11.1)	.07
II	84 (35.1)	22 (55.0)	4 (40.0)	58 (30.7)	
III	86 (36.0)	10 (25.0)	4 (40.0)	72 (38.1)	
IV	43 (18.0)	4 (10.0)	1 (10.0)	38 (20.1)	
No. of tumors					
2	206 (86.2)	31 (77.5)	10 (100)	165 (87.3)	.10
3	22 (9.2)	5 (12.5)	0	17 (9.0)	
>3	11 (4.6)	4 (10.0)	0	7 (3.7)	
Treatment					
Neoadjuvant chemotherapy	34 (14.2)	3 (7.5)	2 (20.0)	29 (15.3)	.38
Adjuvant chemotherapy	120 (50.2)	18 (45.0)	4 (40.0)	98 (51.9)	
Histologic type					
Well differentiated	25 (10.5)	4 (10.0)	0	21 (11.1)	.59
Moderately differentiated	156 (65.3)	17 (42.5)	7 (70.0)	132 (69.8)	
Poor differentiated	58 (24.3)	19 (47.5)	3 (30.0)	36 (19.0)	

Abbreviations: dMMR, deficient mismatch repair; pMMR, proficient mismatch repair.

### MSI-H Ratio in sMPCC vs SPCRC

We retrospectively analyzed the data of patients with SPCRC who underwent surgery and tested their MMR status in our center during the same period. The incidence of dMMR in patients with sMPCC was significantly higher than that in patients with SPCRC (50 of 239 patients vs 872 of 13 037 patients). The results of MSI status detected by NGS were consistent with those of MMR, with incidences of 17 of 78 patients and 5 of 94 patients, respectively (Table 2).

**Table 2.** Differences in MMR and MSI Status Between sMPCC and SPCRC

Characteristic	Patients, No.		*P* value
	sMPCC (n = 239)	SPCRC (n = 13 037)	
MMR status			
All dMMR/dMMR and pMMR	50	872	<.001
All pMMR	189	12 165	
MSI status			
MSI-H/MSI-H and MSS	17	5	.002
All MSS	61	89	

Abbreviations: dMMR, deficient mismatch repair; MMR, mismatch repair; MSI, microsatellite instability; MSI-H, microsatellite instability-high; MSS, microsatellite stability; pMMR, proficient mismatch repair; sMPCC, synchronous multiple primary colorectal cancer; SPCRC, single primary colorectal cancer.

### Somatic Variations in sMPCC vs SPCRC

Among 189 patients who underwent NGS (158 tumors in 78 patients with sMPCC and 111 tumors in 111 patients with SPCRC), we found that the most enriched gene variant type in patients with sMPCC was C>T (G>A) (eFigure 2 in Supplement 1), and the most frequent variant genes were *APC* (103 of 158 tumors [65%]), *KRAS* (78 of 158 tumors [46%]), *TP53* (49 of 158 tumors [31%]), *PIK3CA* (39 of 158 tumors [25%]), and *EGFR* (36 of 158 tumors [23%]), whereas the most frequent variant genes in patients with SPCRC were *APC* (79 of 111 tumors [71%]), *TP53* (71 of 111 tumors [64%]), *KRAS* (44 of 111 tumors [40%]), *FBXW7* (22 of 111 tumors [20%]), and *PIK3CA* (14 of 111 tumors [13%]). We also found that variant genes were significantly different among the 3 subgroups. The top 5 variant genes in the MSI-H group were *APC* (15 of 22 tumors [68%]), *FAT4* (14 of 22 tumors [64%]), *TCF7L2* (13 of 22 tumors [59%]), *KMT2B* 12 of 22 tumors [55%]), and *ARID1A* (10 of 22 tumors [45%]); those in the MSI-H and MSS group were *APC* (8 of 14 tumors [57%]), *KMT2B* (6 of 14 tumors [43%]), *KMT2C* (6 of 14 tumors [43%]), *ATM* (6 of 14 patients [43%]), and *PRKDC* (6 of 14 tumors [43%]); and those in the MSS group were *APC* (80 of 122 tumors [66%]), *KRAS* (60 of 122 tumors [49%]), *TP53* (44 of 122 tumors [36%]), *PIK3CA* (26 of 122 tumors [21%]), and *EGFR* (25 of 122 tumors [20%]) (eFigure 3A in Supplement 1).

The enriched pathways for each lesion in each group of patients with sMPCC and SPCRC are shown in eFigure 3B in Supplement 1. We evaluated 10 canonical signaling pathways with frequent genetic alterations, starting with key cancer genes explored in these pathways in previous TCGA publications, and focused on pathway members likely to be functional contributors or therapeutic targets. The pathways analyzed were (1) cell cycle, (2) Hippo signaling, (3) Myc signaling, (4) Notch signaling, (5) oxidative stress response/NRF2, (6) PI-3-Kinase signaling, (7) receptor-tyrosine kinase (RTK)/RAS/MAP-kinase signaling, (8) TGFβ signaling, (9) P53, and (10) β-catenin/Wnt signaling.^18^ For each group, we computed the fraction of samples with at least 1 alteration in each of the 10 signaling pathways. Fraction genomes altered per group are also provided. The RTK-RAS pathway was the signaling pathway with the highest frequency of alterations across all 4 groups. The other 9 signaling pathways with high alteration frequencies of the most commonly altered genes were slightly different among the 4 groups, but no obvious differences were observed.

### Germline Variations in Patients With sMPCC

Of the 78 patients with sMPCC as detected by sequencing analysis, 15 (19.2%) had pathogenic or likely pathogenic germline variations (PGVs), and 11 (13.75%) had SPCRC. Patients with PGVs were comparable between the sMPCC and SPCRC groups (eTable 2 in Supplement 1). In addition, the associations between PGVs and family history in patients were analyzed and are listed in eTable 3 in Supplement 1. The proportion of patients with family history in the group with PGVs (7 of 15 patients [46.7%]) was significantly higher than that in the group without PGVs (3 of 63 patients [4.8%]).

### TMB in sMPCC vs SPCRC

**Figure 3.**
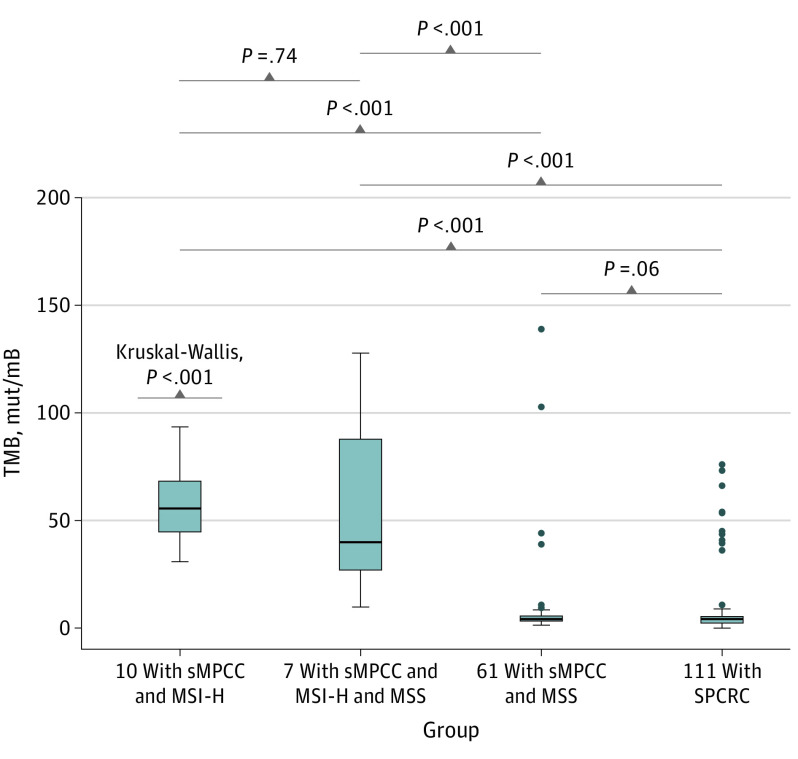
Tumor Mutation Burden (TMB) Value Comparison Among Each Group of Patients With Synchronous Multiple Primary Colorectal Cancer (sMPCC) and Single Primary Colorectal Cancer (SPCRC) Box plot showing TMB value comparison among each group of patients with sMPCC (all microsatellite instability-high [MSI-H], MSI-H and microsatellite stability [MSS], and all MSS) and patients with SPCRC. The TMB value of each patient with sMPCC was determined to be the highest TMB value of each lesion of the patient. A Kruskal-Wallis test was used, and the *P* value was is labeled. The lower and upper box boundaries represent 25th and 75th percentiles, horizontal lines within boxes represent medians, whiskers extend to extreme values ≤1.5 times the IQR, and points beyond whiskers are outliers. mut/mB indicates mutations per megabase.

TMB data were available for 189 patients. Among the 78 patients with sMPCC, the median (IQR) TMB values were 55.6 (44.7-68.3) mutations per megabase among the 10 patients in the MSI-H group, 39.9 (27.0-87.8) mutations per megabase among the 7 patients in the MSI-H and MSS group, and 4.2 (3.3-5.6) mutations per megabase among the 61 patients in the MSS group. On the basis of the TMB value (cutoff = 10 mutations per megabase), all tumors harboring high TMB (TMB-H) were identified in the MSI-H subgroup (10 of 10 patients [100%]), 6 of 7 patients (85.7%) with TMB-H in the MSI-H and MSS subgroups, and 5 of 61 patients (8.2%) with TMB-H in the MSS subgroup (Figure 3). However, among 111 patients with SPCRC, the median (IQR) TMB was 4.2 (2.3-5.4) mutations per megabase, which was similar to the subgroup of all MSS in sMPC. There were no significant differences in patients with TMB-H between the subgroups of all MSS in sMPCC and SPCRC (5 of 61 patients vs 11 of 111 patients). Higher TMB was associated with higher MSI in patients with sMPCC than in those with SPCRC (eTable 4 in Supplement 1).

### Survival Differences Among the Subtypes in sMPCC

Patients with dMMR had a median OS of 95.7 months vs 103.7 months in patients with all pMMR (hazard ratio [HR], 0.995; 95% CI, 0.454-2.180; *P* = .99). Similarly, patients with dMMR had a median PFS of 95.2 months vs 103.3 months in patients with all pMMR (HR, 0.62; 95% CI, 0.35-1.11; *P* = .16). Although no statistically significant difference in OS and PFS was observed between the 2 groups according to MMR status, the trends of OS and PFS were obviously different. A similar trend was observed for MSI status. In patients whose tumors were all MSI-H/MSI-H and MSS, the OS was 95.7 months vs 58.0 months in patients with all MSS (HR, 0.44; 95% CI, 0.17-1.16 months; *P* = .17) (eFigure 4 in Supplement 1).

## Discussion

Owing to the relatively rare incidence of sMPCC in the general population, the profile of pathogenic variations in sMPCC remains unclear. Whether the development of different malignant lesions in the same patient is associated with the same somatic or germline mutation needs to be evaluated. In the past decade, immunotherapies such as PD-1 blockade have achieved significant advancements in the treatment of multiple tumors.^19^ Approximately 12% of all patients with CRC have sporadic MSI/dMMR.^20^ PD-1 blockade has been shown to elicit a good response in dMMR/MSI-H CRC and has been recommended as the first-line treatment for metastatic dMMR/MSI-H CRC.^21^ The MMR/MSI status now plays an increasingly important role in the treatment of CRC; however, whether the MMR/MSI status is consistent in different lesions of the same patient with sMPCC remains unclear. Therefore, in this cohort study, we evaluated the MMR/MSI status of 239 patients with sMPCC among 13 276 patients with CRC treated in the past decade. As expected, the incidence of dMMR/MSI-H in sMPCC was significantly higher than that in SPCRC, as confirmed by IHC and NGS (eTable 1 in Supplement 1). On the basis of these findings, we conclude that patients with sMPCC may have a higher potential benefit from immunotherapy than patients with SPCRC.

To our knowledge, the molecular typing of sMPCC has not been previously reported, this study is the first to evaluate each lesion in all patients with sMPCC, and this study had the largest number of sMPCC cases to date. Interestingly, we found that the MMR/MSI status was not completely consistent among different sMPCC lesions. Some patients with sMPCC might have both dMMR/MSI-H and pMMR/MSS lesions. Thus, we divided the sMPCC into 3 subgroups according to the MMR/MSI status: all dMMR/MSI-H, both dMMR/MSI-H and pMMR/MSS, and all pMMR/MSS groups. The incidence of all pMMR/MSS groups was the highest among the 3 subgroups, which was consistent with the incidence of the pMMR/MSS status in SPCRC. However, the incidence of dMMR/MSI-H in the sMPCC group was significantly higher than that in the SPCRC group.

Although there were no significant differences in OS and PFS between the pMMR/MSS group and the other 2 subgroups, patients with sMPCC with dMMR/MSI-H status tended to have better survival than the pMMR/MSS group, which was because the patients with sMPCC in this cohort had no exposure to immunotherapy, except in the single case we reported. In the context of immunotherapy in CRC^22,23,24,25,26^ and the treatment efficacy of that case, we believe that molecular typing–guided treatment is necessary for patients with sMPCC. For all dMMR/MSI-H subgroups, immunotherapy such as PD-1 blockade may be prioritized. For the dMMR/MSI-H and pMMR/MSS subgroups, PD-1 blockade combined with chemotherapy may be recommended. Neoadjuvant chemotherapy with or without targeted therapy may be suitable for all pMMR/MSS subgroups. Because of the differing variants in the individual lesions of sMPCC and the necessity of molecular typing–guided treatment, we suggest that it is necessary to test the MMR/MSI status of each lesion in patients with newly diagnosed sMPCC.

Genomic analysis revealed that the most enriched gene variant type in patients with sMPCC was C>T (G>A). The incidence of *TP53* variants in sMPCC was significantly lower than that in SPCRC (31% vs 64%). Meanwhile, the incidences of the other most frequently variant genes, including *PIK3CA*, *EGFR*, *ARID1A*, *NF1*, *SOX9*, *FAT4*, and *TCF7L2*, were all higher than those in patients with SPCRC. Moreover, we found that higher TMB was associated with higher MSI in patients with sMPCC than in patients with SPCRC. Furthermore, we found that variant genes differed among the 3 subgroups. The top 5 variant genes were *APC*, *FAT4*, *TCF7L2*, *KMT2B*, and *ARID1A* in the MSI-H group; *APC*, *KMT2B*, *KMT2C*, *ATM*, and *PRKDC* in the MSI-H group; and *APC*, *KRAS*, *TP53*, *PIK3CA*, and *EGFR* in the MSS group. Finally, although we found that patients with pathogenic or likely PGVs were comparable between sMPCC and SPCRC, patients with sMPCC with a family history were more likely to harbor PGVs. In addition, there was no significant difference in pathway enrichment between the different subtypes of sMPCC compared with SPCRC.

### Limitations

The limitations of our study include but are not limited to the lack of sMPCC cases treated with molecular typing–guided treatment. Larger-scale studies and long-term follow-up are needed to further confirm the molecular typing–guided treatment in patients with sMPCC and to correlate the pathological response resulting from this individual strategy with PFS and OS. Prospective trials are necessary to define the role of molecular typing combined with individual therapy in curing and reducing recurrence in patients with sMPCC.

## Conclusions

Our results revealed that the incidence of dMMR/MSI-H in patients with sMPCC was significantly higher than that in patients with SPCRC. We propose that the MMR/MSI status of each lesion in patients with sMPCC should be verified before treatment, and that these patients can be divided into 3 subgroups according to their MMR/MSI status. Our findings indicate that patients with sMPCC with different MMR/MSI statuses may be treated with personalized therapies for better disease management.

## Notes

Supplement 1. **eFigure 1.** Tumor Markers of the sMPCC Case During Treatment and Follow-up **eTable 1.** Differences in MSI/MMR Status Between NGS and IHC in 78 sMPCC Patients **eFigure 2.** Mutation Spectrum in All Lesions of the 78 Patients **eFigure 3.** Landscape of the Top 20 Most Frequently Mutated Genes and Analyses of Pathway Enrichment Comparison Among Each Group of Among sMPCC and SPCRC Groups Patients **eTable 2.** Differences in Pathogenic Germline Variations (PGVs) Between sMPCC and SPCRC Patients **eTable 3.** Correlation of Pathogenic Germline Variations (PGVs) With Family History in sMPCC Patients **eTable 4.** Correlation Between TMB and MSI Status in sMPCC and SPCRC Patients **eFigure 4.** Kaplan-Meier Analysis of OS and PFS by MMR Status (A) and MSI Status (B) in sMPCC Patients

Supplement 2.
Data Sharing Statement
