# Supplementary material for: Molecular Typing and Clinical Characteristics of Synchronous Multiple Primary Colorectal Cancer
Source: JAMA Netw Open. 2022 Nov 23;5(11):e2243457. doi: 10.1001/jamanetworkopen.2022.43457 (PMC9685491; doi:10.1001/jamanetworkopen.2022.43457)
Supplement: Supplement 1. — eFigure 1. Tumor Markers of the sMPCC Case During Treatment and Follow-up eTable 1. Differences in MSI/MMR Status Between NGS and IHC in 78 sMPCC Patients eFigure 2. Mutation Spectrum in All Lesions of the 78 Patients eFigure 3. Landscape of the Top 20 Most Frequently Mutated Genes and Analyses of Pathway Enrichment Comparison Among Each Group of Among sMPCC and SPCRC Groups Patients eTable 2. Differences in Pathogenic Germline Variations (PGVs) Between sMPCC and SPCRC Patients eTable 3. Correlation of Pathogenic Germline Variations (PGVs) With Family History in sMPCC Patients eTable 4. Correlation Between TMB and MSI Status in sMPCC and SPCRC Patients eFigure 4. Kaplan-Meier Analysis of OS and PFS by MMR Status (A) and MSI Status (B) in sMPCC Patients [file jamanetwopen-e2243457-s001.pdf]

Supplemental Online Content

Zhao Y, Wu J, Pei F, et al. Molecular typing and clinical characteristics of synchronous multiple primary colorectal cancer. *JAMA Netw Open*. 2022;5(11):e2243457. doi:10.1001/jamanetworkopen.2022.43457

eFigure 1. Tumor Markers of the sMPCC Case During Treatment and Follow-up
eTable 1. Differences in MSI/MMR Status Between NGS and IHC in 78 sMPCC Patients
eFigure 2. Mutation Spectrum in All Lesions of the 78 Patients
eFigure 3. Landscape of the Top 20 Most Frequently Mutated Genes and Analyses of Pathway Enrichment Comparison Among Each Group of Among sMPCC and SPCRC Groups Patients
eTable 2. Differences in Pathogenic Germline Variations (PGVs) Between sMPCC and SPCRC Patients
eTable 3. Correlation of Pathogenic Germline Variations (PGVs) With Family History in sMPCC Patients
eTable 4. Correlation Between TMB and MSI Status in sMPCC and SPCRC Patients
eFigure 4. Kaplan-Meier Analysis of OS and PFS by MMR Status (A) and MSI Status (B) in sMPCC Patients

This supplemental material has been provided by the authors to give readers additional information about their work.

© 2022 Zhao Y et al. *JAMA Network Open.*

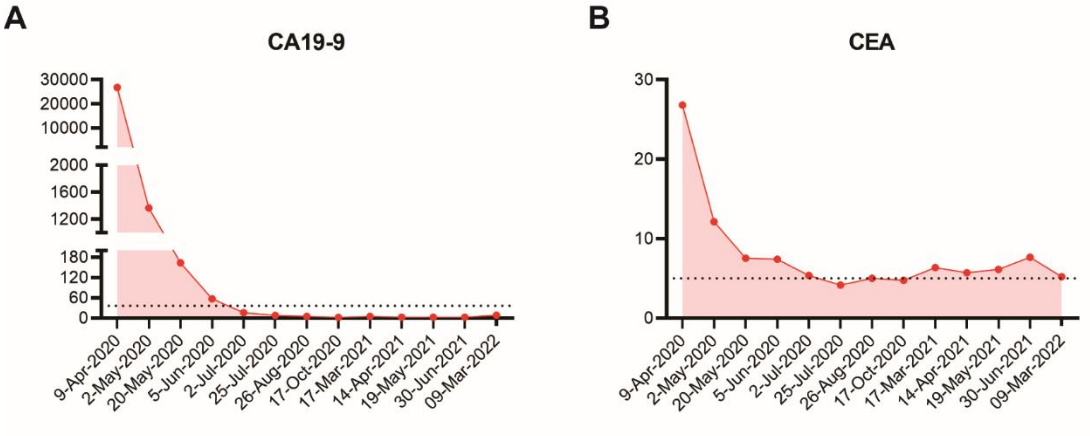

eFigure 1. Tumor Markers of the sMPCC Case During Treatment and Follow-up

The dotted line is the normal value of tumor markers.

CA19-9, carbohydrate antigen 19-9; CEA, carcinoembryonic antigen.

© 2022 Zhao Y et al. *JAMA Network Open.*

eTable 1. Differences in MSI/MMR Status Between NGS and IHC in 78 sMPCC Patients

**Characteristic**	**NGS**	**IHC**
**MSI/MMR status**		
All MSI-H/All dMMR	10	12
MSI-H & MSS/dMMR & pMMR	7	4
All MSS/All pMMR	61	62

sMPCC, synchronous multiple primary colorectal cancer; MMR, mismatch repair; dMMR, mismatch repair; pMMR, proficient mismatch repair; MSI, microsatellite instability; MSI-H, microsatellite instability-high; MSS, microsatellite stability.

© 2022 Zhao Y et al. *JAMA Network Open.*

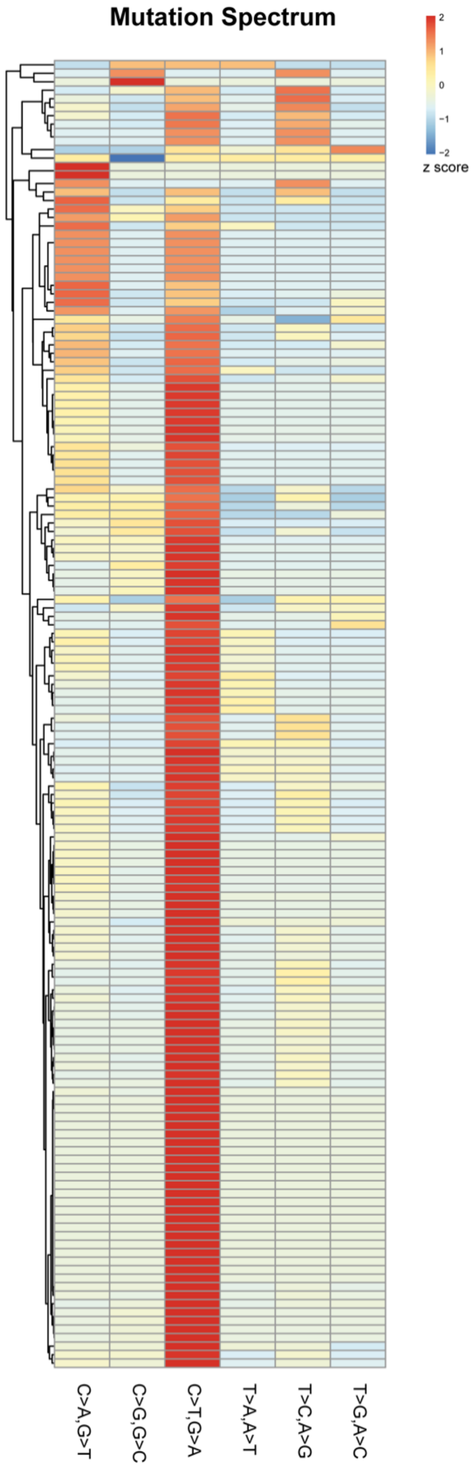

eFigure 2. Mutation Spectrum in All Lesions of the 78 Patients

The mutation spectrum of all samples from sMPCC patients was analyzed and visualized with a heatmap. A deeper red color indicates more enrichment of a specific mutation type.

© 2022 Zhao Y et al. *JAMA Network Open.*

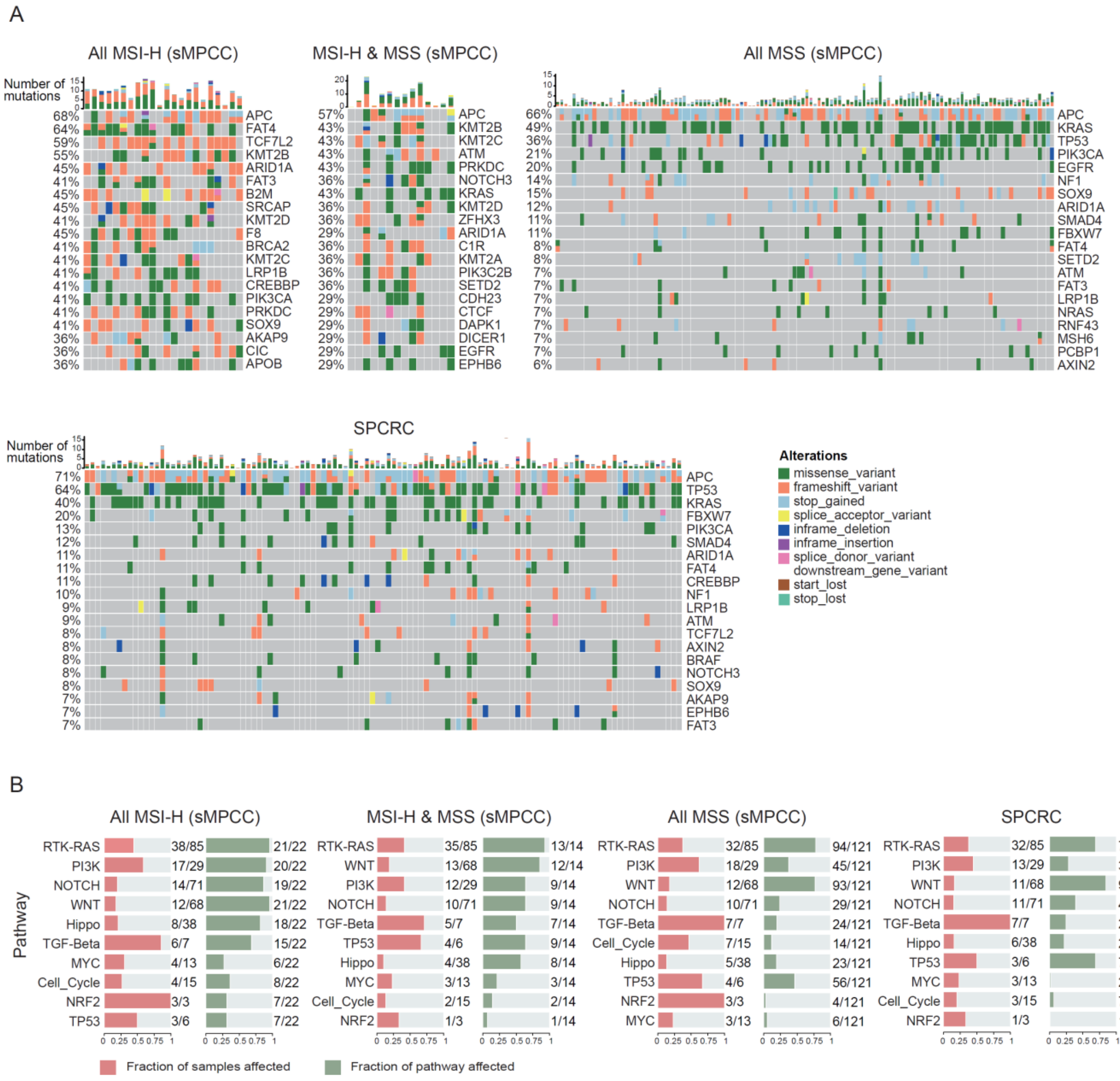

eFigure 3. Landscape of the Top 20 Most Frequently Mutated Genes and Analyses of Pathway Enrichment Comparison Among Each Group of Among sMPCC and SPCRC Groups Patients

A. The top 20 most frequently mutated genes are shown in the histogram on the right of each group of sMPCC patients (all MSI-H, MSI-H&MSS, all MSS) and SPCRC patients. The number of mutations is illustrated in the upper panel. The types of mutations are colored differentially and labeled.

B. The enriched pathways are shown for each lesion among each group of sMPCC patients (all MSI-H, MSI-H & MSS, all MSS) and SPCRC patients. The fraction of mutated genes involved in each pathway is displayed on the left side of each group of sMPCC patients (all MSI-H, MSI-H & MSS, all MSS) and SPCRC patients. The fraction of samples with mutated genes in each pathway is shown on the right side of each group.

© 2022 Zhao Y et al. *JAMA Network Open.*

eTable 2. Differences in Pathogenic Germline Variations (PGVs) Between sMPCC and SPCRC Patients

Characteristic	sMPCC	SPCRC	*P*-value
**Germline mutations**			0.40
With PGVs	15	11	
Without PGVs	63	69	

sMPCC, synchronous multiple primary colorectal cancer; SPCRC, single primary colorectal cancer.

© 2022 Zhao Y et al. *JAMA Network Open.*

eTable 3. Correlation of Pathogenic Germline Variations (PGVs) With Family History in sMPCC Patients

Characteristic	With PGVs	Without PGVs	*p*-value
**Family history**			< 0.001
With family history	7	3	
Without family history	8	60	

sMPCC, synchronous multiple primary colorectal cancer.

© 2022 Zhao Y et al. *JAMA Network Open.*

eTable 4. Correlation Between TMB and MSI Status in sMPCC and SPCRC Patients

Characteristic	sMPCC			SPCRC
	All MSI-H	MSI-H & MSS	All MSS	
TMB-H	10	6	5	11
TMB-L	0	1	56	100

sMPCC, synchronous multiple primary colorectal cancer; SPCRC, single primary colorectal cancer; MSI, microsatellite instability; TMB, tumor mutation burden.

© 2022 Zhao Y et al. *JAMA Network Open.*

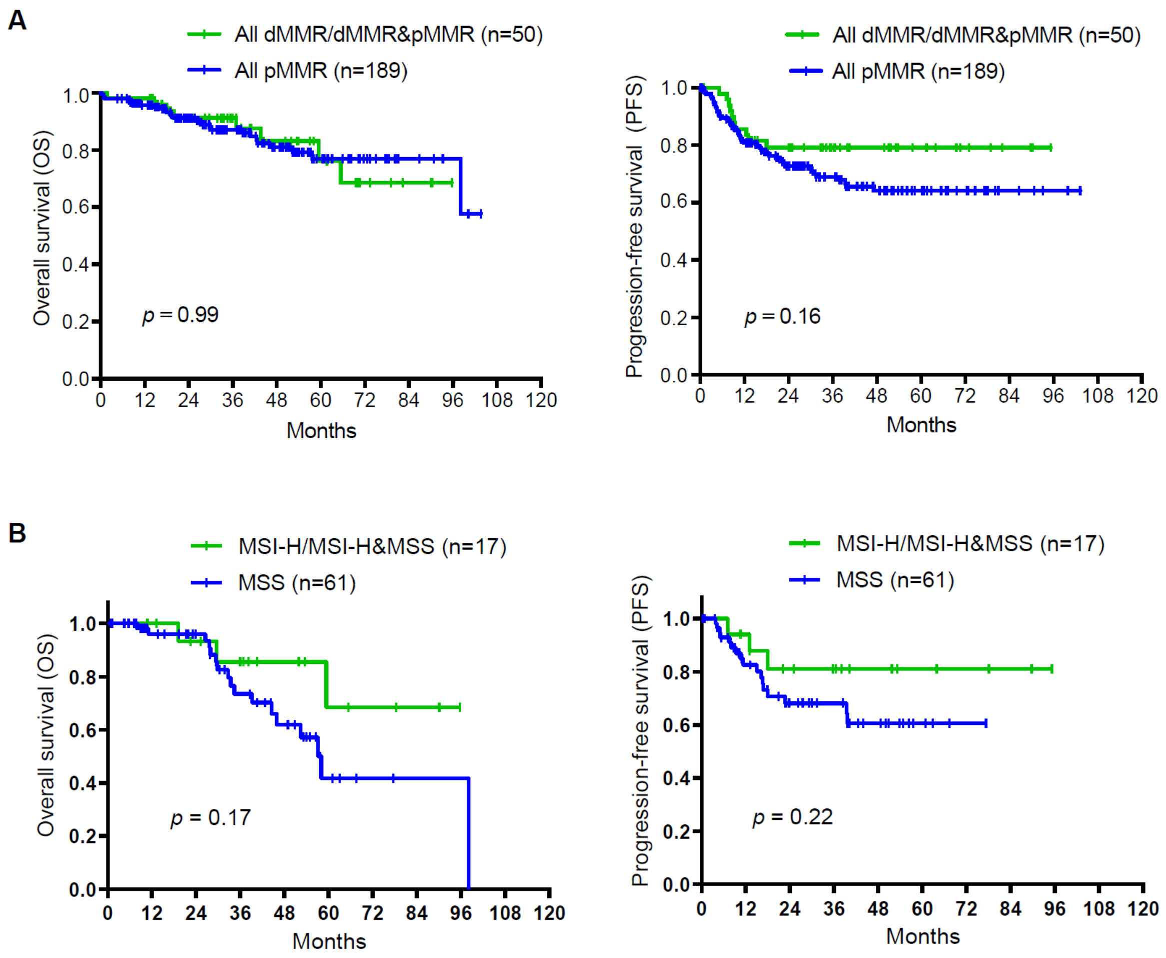

eFigure 4. Kaplan-Meier Analysis of OS and PFS by MMR Status (A) and MSI Status (B) in sMPCC Patients

OS, overall survival; PFS, progression-free survival.

© 2022 Zhao Y et al. *JAMA Network Open.*
