# Supplementary material for: Molecular Typing and Clinical Characteristics of Synchronous Multiple Primary Colorectal Cancer
Source: JAMA Netw Open. 2022 Nov 23;5(11):e2243457. doi: 10.1001/jamanetworkopen.2022.43457 (PMC9685491; doi:10.1001/jamanetworkopen.2022.43457)
Supplement: Supplement 2. — Data Sharing Statement [file jamanetwopen-e2243457-s002.pdf]

Data Sharing Statement

Zhao. Molecular Typing and Clinical Characteristics of Synchronous Multiple Primary Colorectal Cancer. *JAMA Netw Open*. Published November 23, 2022. doi:10.1001/jamanetworkopen.2022.43457

Data
Data available: Yes
Data types: Data (not involving human participants), Data dictionary
How to access data: The data used and/or analyzed during the current study are available from the corresponding author Dr.Jun Huang(huangj97@mail.sysu.edu.cn) upon reasonable request.
When available: With publication

Supporting Documents
Document types: Statistical/analytic code
How to access documents: The statistical/analytic code are available from the corresponding author Dr.Jun Huang(huangj97@mail.sysu.edu.cn) upon reasonable request.
When available: With publication

Additional Information
Who can access the data: The data will be available to researchers whose proposed use of the data has been approved.
Types of analyses: The data will be made available for a scientific research purpose.
Mechanisms of data availability: The data will be made available after approval of a proposal and with a signed data access agreement.
Any additional restrictions: no
